# Analyzing COVID-19 Vaccination Behavior Using an SEIRM/V Epidemic Model With Awareness Decay

**DOI:** 10.3389/fpubh.2022.817749

**Published:** 2022-01-27

**Authors:** Chao Zuo, Fenping Zhu, Yuting Ling

**Affiliations:** School of Management Engineering and E-Commerce, Zhejiang Gongshang University, Hangzhou, China

**Keywords:** COVID-19, epidemic model, awareness spreading, awareness fading, vaccination behaviors

## Abstract

Information awareness about COVID-19 spread through multiple channels can stimulate individuals to vaccinate to protect themselves and reduce the infection rate. However, the awareness individuals may lose competency over time due to the decreasing quality of the information and fading of awareness. This paper introduces awareness programs, which can not only change people from unaware to aware state, but also from aware to unaware state. Then an SEIRM/V mathematical model is derived to study the influence of awareness programs on individual vaccination behavior. We evaluate the dynamical evolution of the system model and perform the numerical simulation, and examine the effects of awareness transformation based on the COVID-19 vaccination case in China. The results show that awareness spread through various information sources is positively associated with epidemic containment while awareness fading negatively correlates with vaccination coverage.

## Introduction

Currently, the novel coronavirus disease (COVID-19) is spreading globally as a huge health hazard and causing widespread public concern (1, 2). Information awareness of COVID-19 can stimulate individuals to adopt spontaneous protective behaviors, such as washing hands, wearing masks, social distancing, vaccination, etc., which plays a very important role in controlling disease outbreaks (3, 4). Therefore, the development of formal models to research the mutual effect between disease transmission and information-aware behavioral responses is receiving increasing attention (5, 6).

In modeling the effects of awareness on the dissemination of epidemic and its control, there are two notable approaches been used to include information awareness into the framework of the epidemic model. The first approach, usually represented by an exponential function, is to directly combine the impact of information into the transmission rate of the epidemic, which in turn reduces disease transmission as a result of disease awareness. For example, Zuo et al. (7) proposed a two-layer UAU-SIR (unaware-aware-unaware/susceptible-infected-recovered) model with neighbor behavior on multiplex networks to study the mutual effect between epidemics dissemination and awareness diffusion, and examined the impact of these intervening measures ground on modeling of the awareness-infectious disease and the data of COVID-19 transmission case. Zhao et al. (8) developed a SEIR/V-UA (susceptible–vaccinated–exposed–infected–recovered with unaware–aware) model to explore the joint impact of the awareness diffusion and epidemic transmission, and verified the model through the Monte Carlo (MC) method and numerical simulation on the scale-free networks. Li et al. (9) studied the impact of global and the local awareness on the dynamics of an SIR epidemic, and validated the infection rate and network degree distribution determine the scale of the effect. Ye et al. (10) proposed a heterogeneous disease-information-behavior propagation model to study how various types of individuals (overreacting vs. underreacting) affect the epidemic outbreak and the prevalence of protective behavior, and performed the numerical simulation to research the impact of the different on the epidemic. The second approach is to develop a separate compartment representing the level of epidemic awareness within the population. Hence, transitions between the classes of unaware and aware individuals within the population depend on the level of awareness in circulation. For example, Teslya et al. (11) established a deterministic compartmental model to study COVID-19 propagation in a population stratified by epidemic status (aware and unaware), and conducted sensitivity analyses in regard to the time delay from diagnosis to isolation of infected individuals. Misra et al. (12) proposed a susceptible–infected–susceptible (SIS) model to explore the influence of awareness programs actuated by the media on the diffusion of epidemic, and found the awareness prompts some susceptible to quarantine themselves (12). Saha et al. (13) proposed an SEIRS compartmental model on COVID19 transmission which explains the impact of information about appropriate preventive measures on an individual's behavioral response. Agaba et al. (14) proposed a susceptible–infected–recovered–susceptible (SIRS) with time-delayed model to research the effect of awareness information on vaccination, and analyzed the feasibility and stability analysis of disease-free and endemic equilibria, as well as the conditions for endemic steady-state Hopf bifurcation. Zhou et al. (15) introduced a dynamic compartmental model incorporating the awareness programs as a separate compartment to study the interplay between disease spreading and the media reports, and found media report can be regarded as an efficient way to alleviate the COVID-19 transmission during the primary stage of an outbreak. Obviously, awareness spread through multiple channels can provoke individuals to adopt spontaneous protective behaviors to reduce their chances of becoming infected.

Recently, many countries enter epidemic controlled normalization process with no significant declining COVID-19 cases, the widespread use of COVID-19 vaccines is the most effective way prevent substantial morbidity and mortality (16). Information awareness can influence an individual's vaccination decisions on whether or not to be vaccinated can play a critical role in achieving sufficient and sustained vaccination coverage (17–19). However, awareness individuals may lose competency (self-protection) over time due to the decreasing quality of the information and fading of awareness. For example, China, where the first case of COVID-19 was detected on Dec. 31, 2019, has entered a “controlled normalization process” after 1 year with strict measures. Some individuals thought ‘not many people contract the disease, so the chances are low for me too’, they didn't think they need injecting vaccines again to protect themselves to be unawareness individuals, resulting in the low vaccination rates in mid-February, 2021.

Motivated by the above considerations, we introduce the awareness programs that people not only alter their state from unaware to aware, but also from aware to unaware state, and propose a compartmental model to analyze the influence of awareness programs on individual vaccination behavior.

This paper is organized as follows. Section Model derives the model. Section Basic Reproduction Number and Possible Equilibria deduces the basic reproduction number and possible equilibria. Section Numerical Simulation presents the numerical simulations and analyze the decision behavior of COVID-19 vaccines and epidemic size. Section Conclusion shows the conclusions.

## Model

**Figure 1:**
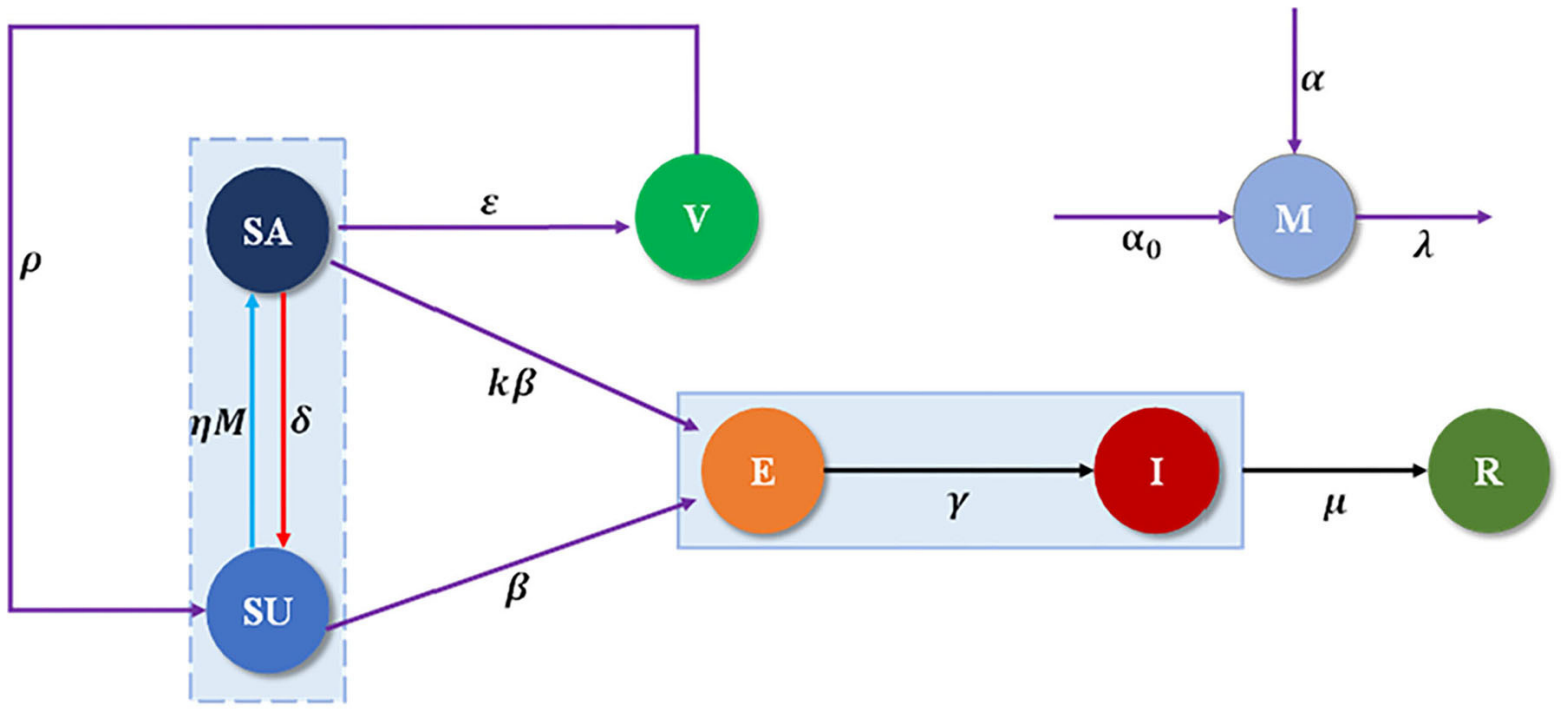
Schematic of SEIRM/V dynamics in multilayer networks.

The total population *N* individuals are separated into seven compartments (*SEIRM/V*), including unaware-susceptible (*S*_*u*_), aware-susceptible (*S*_*a*_), infectious without symptoms (or exposed *E*), infectious with symptoms (or infected *I*), recovered (*R*) and vaccinated (*V*), *M(t)* shows the accumulated density of awareness programs driven by information sources, which consists of three parts, as shown in Figure 1. The α represents the rate of awareness arising from the aware neighbors (e.g., local prevalence), α_0_ is the response intensity of awareness programs on the number of new cases detected, and λ is the waning rate of information due to the decreasing quality of the information. Unaware individuals develop into aware at the awareness transmission rate η*M*, if s/he is in possession of disease-related awareness from global epidemic information η and *M(t)*, and aware individuals become unaware with probability δ, representing that an individual would lose alertness of the disease with time. Each unaware-susceptible become exposed at the disease transmission rate β. Aware-susceptible may become exposed at the rate *kβ*, where *1-k* defines the degree to which intermediate protection measures taken with awareness to decrease the possibility of infection, while aware-susceptible could develop into vaccinated individuals (*V*) to be protected with probability ε. Then by passing through potential and incubation periods in which the rate from the exposed state to infected state is γ, clinical characteristics of the undiagnosed infected cases, begin to appear and enter them into the confirmed infected compartment (*I*). Confirmed infected persons might recuperate from COVID-19 and enter into the recovered compartment (*R*) with the recovery rates of μ. Furthermore, it is supposed that the individuals lose their immunity against the epidemic after a period of time *1/*ρ. See more detailed definitions of variables and parameters listed in Table 1.

**Table 1:** Description and baseline values of the parameters of model (1).

**Parameter**	**Meanings**	**Baseline value**	**Reference**
β	Transmission rate	0.468	(20)
γ	Fraction of exposed class who become infective	0.1818	(21)
*k*	Fraction of reduction in susceptibility to infection due to being aware	0.3	Estimated
	Growth rate of disease awareness from the reported number of infections	0.2	(14)
α	Growth rate of local disease awareness arising from aware neighbors	0.3	(14)
λ	Decay rate of information due to the decreasing quality of the information	0.3	(22)
μ	Recovery rate for infected individual	0.278	(23)
η	Growth rate of disease awareness arising from global source	0.1	Estimated
δ	Rate of loss of awareness in susceptible individuals	0.1	(14)
ε	Fraction of aware individuals who are vaccinated	0.04	Estimated
ρ	Rate of losing vaccine immunity	0.05	Estimated

The evolution of individuals through the *SEIRM/V* model with awareness programs driven vaccination is modeled with the next set of ordinary differential equations:


(1) $$\begin{array}{l} \{\begin{array}{ccc} \frac{dS_{u}}{dt} & = & \frac{\beta S_{u}I}{N}-\eta MS_{u}+\delta S_{a}+\rho V\;\\ \frac{dS_{a}}{dt} & = & \;\frac{k\beta S_{a}I}{N}+\eta MS_{u}-\delta S_{a}-\varepsilon S_{a}\;\\ \frac{dE}{dt} & = & \frac{\beta S_{u}I}{N}+\frac{k\beta S_{a}I}{N}-\gamma \text{E }\\ \frac{dI}{dt} & = & \;\gamma \text{E}-\mu I\;\\ \frac{dR}{dt} & = & \mu I\;\\ \frac{dV}{dt} & = & \varepsilon S_{a}-\;\rho V\;\\ \frac{dM}{dt} & = & \frac{\alpha S_{a}}{N}+\alpha_{0}I-\lambda M\; \end{array} \end{array}$$

where *S*_u_(0) > 0, *S*_a_(0) ≥ 0, *V*(0) ≥ 0, *E*(0) ≥ 0, *I*(0) ≥ 0, *R*(0) ≥ 0, *M*(0) ≥ 0, is the initial conditions.

For the analysis of model (1), we need the region of attraction (24) which is given by the set:


$$\begin{array}{l} \Omega =\{\left(S_{u},S_{a},V,E,I,R,M\right)\in {ℜ}_{+}^{7},0\leq S_{u},S_{a},V,E,I,R\\ \leq N,0\leq M\leq \tilde{M}\} \end{array}$$

where $\tilde{M}=\max\left\{M_{0},\;\frac{\alpha +\alpha_{0}}{\lambda }\;\right\}$

## Basic Reproduction Number and Possible Equilibria

The above model system (1) has two non-negative equilibria:

(i) Disease free equilibrium *E*_1_(N, 0, 0, 0, 0, 0, 0)
(ii) Disease free equilibrium $E_{2}\left(\frac{N\lambda \left(\delta +\epsilon \right)}{\alpha \eta },0,0,0,\frac{-N\left(\delta \lambda -\alpha \eta +\varepsilon \lambda \right)}{\alpha \eta },0,0\right)$

We derive the basic reproduction number *R*_0_ for the epidemic model using the next-generation method (25). We define two matrices, *F* and *Q*, where *F* (for the emerging infection terms) is the pertinent non-negative matrix and *Q* (for the residual transfer terms) is the non-singular M-matrix, are given, respectively, by


$$\begin{array}{r} F=\left(\begin{array}{cc} 0\; & \frac{\beta S_{u}}{N}+\frac{k\beta S_{a}}{N}\\ 0\; & 0\\ \; \end{array}\right)\\ \text{and }Q=\left(\begin{array}{c} \gamma \;0\\ -\gamma \;\mu \end{array}\right) \end{array}$$

The control reproduction number, represented by *R*_*V*_, is then provided by $R_{V}=\rho \left(FQ^{-1}\right)$, where ρ is the spectral radius of the matrix *FQ*^−1^. It follows that


$$\begin{array}{l} R_{V}=\frac{\lambda \left(\delta +\varepsilon \right)}{\alpha \eta }R_{0}, \end{array}$$

Where $R_{0}=\frac{\beta }{\mu }$ is the basic reproduction number lack of vaccination. The quantity *R*_*V*_ gauges the average number of new infections attribute to a typical infectious individual among some susceptible individuals who are vaccinated (26, 27). It is worth mentioning that the threshold quantity*R*_*V*_ < 1, since ${\frac{\lambda \left(\delta +\epsilon \right)}{\alpha \eta }R}_{0}<1$. Both *R*_0_ and *R*_*V*_ serve to measure the severity of an epidemic.

The Jacobian matrix corresponding to the system (1) as shown below,


(2) $$\begin{array}{l} J=\left(\begin{array}{lllllll} -\eta M-\frac{\beta I}{N} & \delta & 0 & \frac{-\beta S_{\text{u}}}{N} & 0 & \rho & -\eta S_{u}\\ \eta M & -\delta -\varepsilon -\frac{k\beta I}{N} & 0 & \frac{-k\beta S_{a}}{N} & 0 & 0 & \eta S_{u}\\ \frac{\beta I}{N} & \frac{k\beta I}{N} & -\gamma & \frac{\beta S_{\text{u}}}{N}+\frac{k\beta S_{a}}{N} & 0 & 0 & 0\\ 0 & 0 & \gamma & -\mu & 0 & 0 & 0\\ 0 & 0 & 0 & \mu & 0 & 0 & 0\\ 0 & \varepsilon & 0 & 0 & 0 & -\rho & 0\\ 0 & \frac{\alpha }{N} & 0 & \alpha_{0} & 0 & 0 & -\lambda \end{array}\right) \end{array}$$

We calculated the Jacobian matrix at *E*_1_ and get its corresponding characteristic equation to establish the local stability of the infection-free equilibrium:


(3) $$\begin{array}{l} J\left(E_{1}\right)=\left(\begin{array}{lllllll} 0 & \delta & 0 & -\beta & 0 & \rho & -\eta N\\ 0 & -\delta -\varepsilon & 0 & 0 & 0 & 0 & \eta N\\ 0 & 0 & -\gamma & \beta & 0 & 0 & 0\\ 0 & 0 & \gamma & -\mu & 0 & 0 & 0\\ 0 & 0 & 0 & \mu & 0 & 0 & 0\\ 0 & \varepsilon & 0 & 0 & 0 & -\rho & 0\\ 0 & \frac{\alpha }{N} & 0 & \alpha_{0} & 0 & 0 & -\lambda \end{array}\right) \end{array}$$

$$\begin{array}{l} \Phi \left(L\right)=(L-(\frac{\sqrt{\delta^{2}+\varepsilon^{2}+\lambda^{2}+2\delta \varepsilon -2\delta \lambda -2\varepsilon \lambda +4\alpha \eta }}{2}\\ -\frac{\delta }{2}-\frac{\varepsilon }{2}-\frac{\lambda }{2}))\\ \left(L+\left(\frac{\delta }{2}+\frac{\varepsilon }{2}+\frac{\lambda }{2}+\frac{\sqrt{\delta^{2}+\varepsilon^{2}+\lambda^{2}+2\delta \varepsilon -2\delta \lambda -2\varepsilon \lambda +4\alpha \eta }}{2}\right)\right)\\ \left(L-\left(\frac{\sqrt{\gamma^{2}-2\gamma \mu +4\beta \gamma +\mu^{2}}}{2}-\frac{\gamma }{2}-\frac{\mu }{2}\right)\right)\\ \left(L+\left(\frac{\gamma }{2}+\frac{\mu }{2}+\frac{\sqrt{\gamma^{2}-2\gamma \mu +4\beta \gamma +\mu^{2}}}{2}\right)\right)\left(L+\rho \right)\\ L_{1}=-\rho <0,\\ L_{2}=-\frac{\gamma }{2}-\frac{\mu }{2}-\frac{\sqrt{\gamma^{2}-2\gamma \mu +4\beta \gamma +\mu^{2}}}{2}<0\\ L_{3}=\frac{\sqrt{\gamma^{2}-2\gamma \mu +4\beta \gamma +\mu^{2}}}{2}-\frac{\gamma }{2}-\frac{\mu }{2}\\ =\frac{\sqrt{{\left(\gamma +\mu \right)}^{2}+4\gamma \left(\beta -\mu \right)}}{2}-\frac{\left(\gamma +\mu \right)}{2}<0,\\ L_{4}=-\frac{\delta }{2}-\frac{\varepsilon }{2}-\frac{\lambda }{2}\\ -\frac{\sqrt{\delta^{2}+\varepsilon^{2}+\lambda^{2}+2\delta \varepsilon -2\delta \lambda -2\varepsilon \lambda +4\alpha \eta }}{2}<0\\ L_{5}=\frac{\sqrt{\delta^{2}+\varepsilon^{2}+\lambda^{2}+2\delta \varepsilon -2\delta \lambda -2\varepsilon \lambda +4\alpha \eta }}{2}-\frac{\delta }{2}-\frac{\varepsilon }{2}\\ -\frac{\lambda }{2}=\frac{\sqrt{{\left(\varepsilon +\lambda +\delta \right)}^{2}+4\left(\alpha \eta -\delta \lambda -\varepsilon \lambda \right)}}{2}-\frac{\left(\varepsilon +\lambda +\delta \right)}{2}<0 \end{array}$$

It is obvious that both eigenvalues *L*_1_, *L*_2_ and *L*_4_ are negative, while the third and the fifth conditions *L*_3_, *L*_5_ is negative if and only if


(4) $$\begin{array}{l} R_{V}=\frac{\lambda \left(\delta +\varepsilon \right)}{\alpha \eta }R_{0}<1,R_{0}=\frac{\beta }{\mu } \end{array}$$

Moreover, the Jacobian matrix at *E*_2_ and get its corresponding characteristic equation:


(5) $$\begin{array}{l} J\left(E_{2}\right)=\left(\begin{array}{lllllll} 0 & \delta & 0 & \frac{-\lambda \beta \left(\delta +\varepsilon \right)}{\alpha \eta } & 0 & \rho & \frac{-\lambda N\left(\delta +\varepsilon \right)}{\alpha }\\ 0 & -\delta -\varepsilon 0 & 0 & 0 & 0 & 0 & \frac{\lambda N\left(\delta +\varepsilon \right)}{\alpha }\\ 0 & 0 & -\gamma & \frac{\lambda \beta \left(\delta +\varepsilon \right)}{\alpha \eta } & 0 & 0 & 0\\ 0 & 0 & \gamma & -\mu & 0 & 0 & 0\\ 0 & 0 & 0 & \mu & 0 & 0 & 0\\ 0 & \varepsilon & 0 & 0 & 0 & -\rho & 0\\ 0 & \frac{\alpha }{N} & 0 & \alpha_{0} & 0 & 0 & -\lambda \end{array}\right) \end{array}$$

(6) $$\begin{array}{l} \Phi \left(L\right)=(L+(\frac{\gamma }{2}+\frac{\mu }{2}\\ -\frac{\sqrt{\left(\alpha \eta \gamma^{2}+\alpha \eta \mu^{2}+4\beta \delta \gamma \lambda +4\beta \varepsilon \gamma \lambda -2\alpha \eta \gamma \mu \right)/\left(\alpha \eta \right)}}{2})) \end{array}$$

$$\begin{array}{l} (L+(\frac{\gamma }{2}+\frac{\mu }{2}\\ +\frac{\sqrt{\left(\alpha \eta \gamma^{2}+\alpha \eta \mu^{2}+4\beta \delta \gamma \lambda +4\beta \varepsilon \gamma \lambda -2\alpha \eta \gamma \mu \right)/\left(\alpha \eta \right)}}{2}))\\ \left(L+\delta +\varepsilon +\lambda \right)\left(L+\rho \right)\\ L_{1}=-\rho <0\\ L_{2}=-\delta -\varepsilon -\lambda <0\\ L_{3}=-\frac{\gamma }{2}-\frac{\mu }{2}\\ -\frac{\sqrt{\left(\alpha \eta \gamma^{2}+\alpha \eta \mu^{2}+4\beta \delta \gamma \lambda +4\beta \varepsilon \gamma \lambda -2\alpha \eta \gamma \mu \right)/\left(\alpha \eta \right)}}{2}<0\\ L_{4}=\frac{\sqrt{\left(\alpha \eta \gamma^{2}+\alpha \eta \mu^{2}+4\beta \delta \gamma \lambda +4\beta \varepsilon \gamma \lambda -2\alpha \eta \gamma \mu \right)/\left(\alpha \eta \right)}}{2}\\ -\frac{\gamma }{2}-\frac{\mu }{2}\\ =\frac{\sqrt{{\left(\gamma +\mu \right)}^{2}+4\gamma \left(\beta \delta \lambda +\beta \varepsilon \lambda -\alpha \eta \mu \right)/\left(\alpha \eta \right)}}{2}-\frac{\left(\gamma +\mu \right)}{2}<0 \end{array}$$

It is accessible that both eigenvalues *L*_1_, *L*_2_ and *L*_3_ are negative while the fourth conditions *L*_4_ is negative if and only if


$$\begin{array}{l} R_{V}=\frac{\lambda \left(\delta +\varepsilon \right)}{\alpha \eta }<1 \end{array}$$

Hence, the infection-free equilibrium *E*_1_ and *E*_2_ is locally asymptotically stable when *R*_*V*_ <1. What is noteworthy is that the awareness growth rate α_0_ related to the reported confirmed number of infections does not affect the stability of the disease-free steady state. And the causes are as follows: in the neighborhood of the disease-free steady state, if *R*_*V*_ < 1, the number of confirmed infected individuals would tend zero, thus its contribution to the growth of awareness arising from the newly confirmed cases reducing to zero, and therefore, it would have no further influence on the stability of *E*_1_ and *E*_2_.

**Figure 2:**
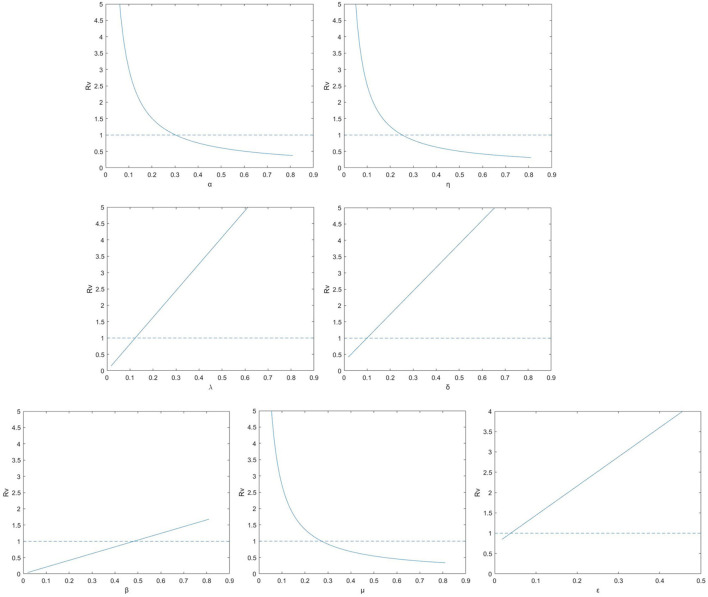
The control reproduction number *R*_*V*_ for model (1) (solid lines) for the relevant model with vaccination according to parameters λ, δ, ε, β, α, η, and μ.

We further explore the impact on the control reproduction number *R*_*V*_ when the parameters variation, see Figure 2. In each subgraph, a line existed to illustrates where *R*_*V*_ crosses the unity. If those parameter values in the graph is below the line, the epidemic can be extinct and where it is beyond the indicator line, the disease can continue to exist (28). It can be observed that *R*_*V*_ is raising for parameters β, λ, δ and ε, while it is decreasing for parameters α, η and μ. Therefore, the disease can be retarded or even removed by controlling the parameters so that *R*_*V*_ locates beneath unity.

## Numerical Simulation

The formerly described model is researched via MC simulations. Considering that vaccine is imperfect and the individuals would lose their immunity against the epidemic after a period of time, we perform simulation on Erdos–Rényi (ER) network with 1,000 nodes and the average degree with <k> = 4. Besides, the nodes whose initial condition is set to be 2% are infected. The rules of iterative coupling dynamic processes are updated in parallel until they converge to the steady state. To evaluate the effect of epidemic spreading, we let ρ^*X*^ (X = S, A, E, I, R, V) present the fraction of the *S*_*u*_, *S*_*a*_, E, I, R, V component in the total nodes.

**Figure 3:**
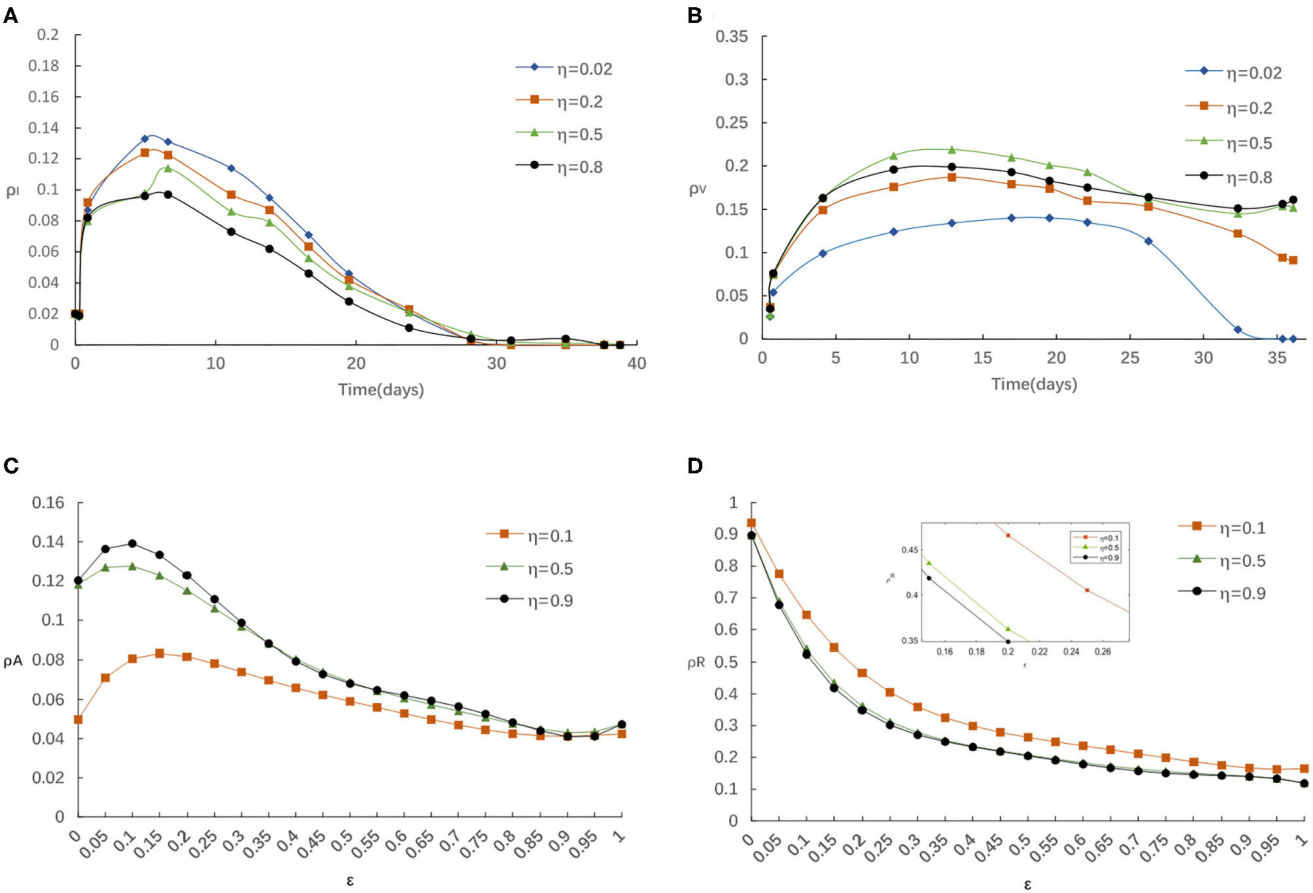
**(A)** The fraction of infected for various η with α = 0.3, α_0_ = 0.2, δ = 0.1. **(B)** The fraction of vaccinated for various η with α = 0.3, α_0_ = 0.2, δ = 0.1. **(C)** The fraction of aware for various η with α = 0.3, α_0_ = 0.2, δ = 0.1. **(D)** The fraction of recovered for various η with α = 0.3, α_0_ = 0.2, δ = 0.1.

First, we evaluate the influence of globe epidemic information η on epidemic spreading. Figure 3 displays the evolution of ρ^*I*^(*t*) and ρ^*V*^(*t*) for four typical η = 0.02, 0.2, 0.5, and 0.8, correspondingly. We find that the peak of ρ^*I*^(*t*) reduces with the increase of η from Figure 3A. People with elevated levels of awareness will be capable of slowing down or stopping the spread of an epidemic by reducing the infectivity and susceptibility of aware individuals. From Figure 3B we notice that the final vaccinated density ρ^*V*^(*t*) will depend on η when the epidemic spreading ends, while, when the epidemic spreading is under way, ρ^*V*^(∞) decreases with the increase of η in the case of relatively larger η. Here we know that the impact of global information always limited even with stronger η. The above result can be account as follows: for larger η, there will be more globe epidemic information appears in the media and thus results in more individuals covered by the awareness during the outbreak. On this occasion, it is accessible for a person to notice its infected neighbors and then get vaccinated. However, individuals can obtain overload information from global news coverage as the government put a higher value on epidemic control. Then, some may not feel the need to self-protection against COVID-19 because they rely heavily on implementation of the series government measures on COVID-19 such as temporary measures to limit and delay the infection rates in COVID-19 through voluntarily isolating, social distancing, wearing masks, taking vaccination.

Meanwhile, we explore the impact of various η on the fraction of aware/recovered individuals as in Figures 3C,D. It is clear that the fraction of aware individuals increases and the fraction of recovered individuals decreases, with the increase of η. Because, individuals should take some protection against infectious after getting information about disease, which broadens the size of aware-susceptible population will decrease not only the recovered but also the infected individuals. On balance, the awareness-driven vaccination still has a positive effect in controlling the epidemic spreading, just heavy reliance on a single global information source is risky.

For example, in mid-April (between 15 March and 15 April, 2021), China had only administered enough doses for just under 2% of its population, while it aimed to vaccinate 40% of its population (or 560 million people) by June. Facing this dilemma, the Chinese government became more active to encourage people to get vaccinated. Thus, with increasing information awareness obtained from government (globe epidemic information increases), individuals changed their vaccination behavior and became more willing to get vaccinated, but surprisingly, the positive effect of global information awareness on vaccination rates was limited because of the lack of local information contributions from aware neighbors, which promoted the ongoing act of letting down their guard and mass gatherings had become the norm again.

**Figure 4:**
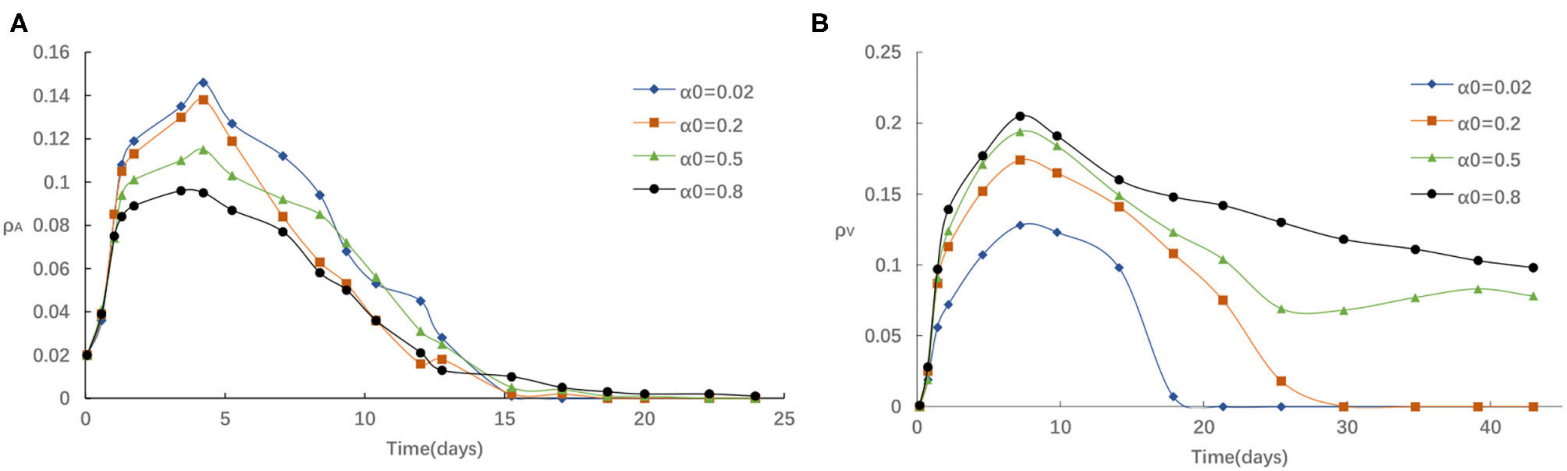
**(A)** The fraction of infected for different α_0_ with η = 0.05, α = 0.3, δ = 0.1. **(B)** The fraction of vaccinated for different α_0_ with η = 0.05, α = 0.3, δ = 0.1.

Second, we study the influence of the rate of awareness arising from the newly confirmed cases α_0_ on epidemic spreading. Figure 4 presents the evolution of ρ^*I*^(*t*) and ρ^*V*^(*t*) for four typical α_0_ = 0.02, 0.2, 0.5 and 0.8, correspondingly. It is accessible to see that Figure 4 is analogous to Figures 3A,B, indicating that the infected density ρ^*I*^ will decrease with the increase of α_0_ and the vaccinated density ρ^*V*^(*t*) will increase with the increase of α_0_. We can explain this phenomenon in a similar way as follows: For a larger α_0_, there is more likely for an individual to take a vaccination once it received strong stimulations of updated information about the reported number of infections, and thus lessen the epidemic spreading. Overall, we see that refusing vaccinations for side effects, the free-riding behavior or other reasons will lead to a higher peak in the ρ^*I*^ curve when the case of both small α_0_ and small η, which indicate that losing awareness will cause more people being affected by the diseases.

For example, in mid-August (between 20 July and 15 August, 2021), a COVID-19 outbreak first discovered in Nanjing has transmission to five provinces and Beijing prompted authorities to institute local lockdowns, prevent people and vehicles from leaving their local areas, close schools, and require residents to get tested for COVID-19. Days later, Jiangsu reported that a lot of people had signed up for vaccines with residents waiting in long lines outside vaccine centers to get injected. The daily dose reached its peak on August 3 (An additional 17.85 million doses were administered in a single day). The number of vaccinations had increased dramatically. This phenomenon can be explained as: the repeated local outbreaks have proved the most effective way to encourage people to get vaccinated. In other words, the accumulated density of awareness programs increases as the number of infected neighbors increases, making people more willing to get vaccinated, thereby further increasing vaccine coverage.

**Figure 5:**
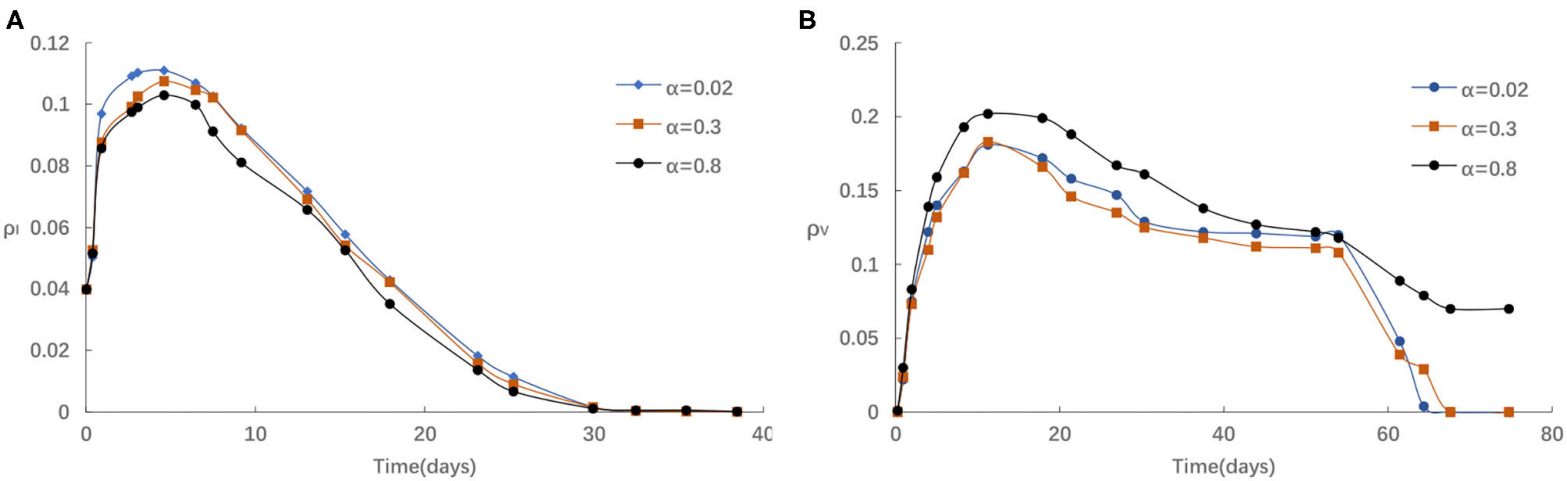
**(A)** The fraction of infected for different α with η = 0.05,α_0_ = 0.2, δ = 0.1. **(B)** The fraction of vaccinated for different α with η = 0.05, α_0_ = 0.2, δ = 0.1.

Third, we investigate the impact of the rate of awareness arising from the aware neighbors α on epidemic spreading. Figure 5 presents the evolution of ρ^*I*^(*t*) and ρ^*V*^(*t*) for three typical α = 0.02, 0.3, and 0.8, respectively. We find that the peak of ρ^*I*^(*t*) reduces with the increase of α from Figure 5A. As in Figure 5B, if α is comparatively small, for instance, α < 0.3, the homologous effect of various α is finite to a large extent because of the case that fraction of aware individuals is not enough. When α is large, people are more likely to react to information around them via imitative behavior with individuals, thus, ρ^*V*^(*t*) will increase much faster than in the case of small α. Overall, aware-susceptible individuals prefer to be vaccinated for more effective protection when large α (for instance, α > 0.3). Especially, when α varies from 0.3 and 0.8, the effect is even more clear. The primary cause is that when there are a lot of awareness owners in the surrounding population, the willingness to acquire awareness will be stronger, hence more people are willing to get vaccinated. Because s/he exhibit herd-like behavior and believes that such awareness is usefulness, may bring benefits, or popular.

**Figure 6:**
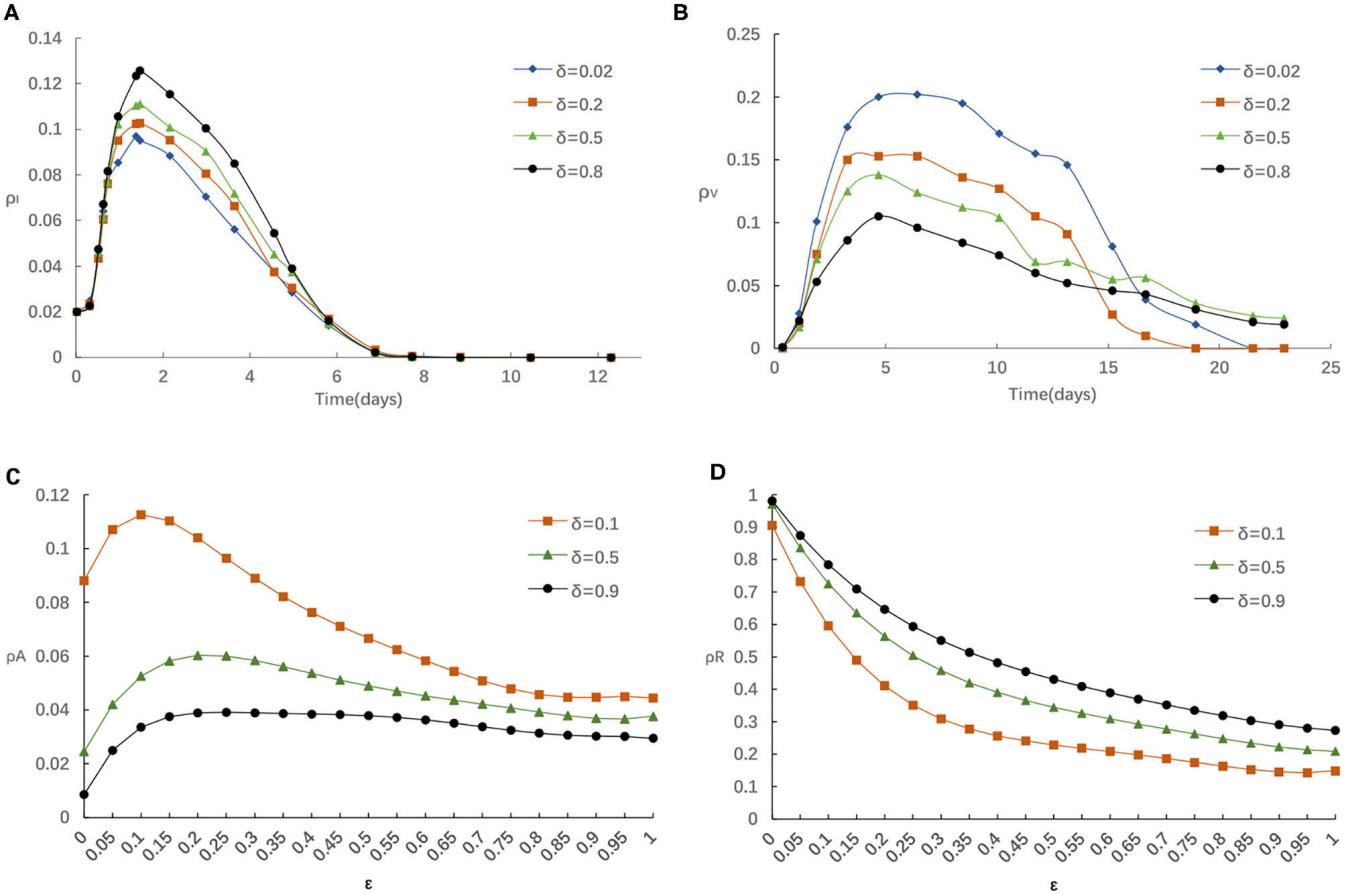
**(A)** The fraction of infected for various δ with η = 0.05,α = 0.3, α_0_ = 0.2. **(B)** The fraction of vaccinated for various δ with η = 0.05, α = 0.3, α_0_ = 0.2. **(C)** The fraction of aware for various δ with η = 0.05, α = 0.3, α_0_ = 0.2. **(D)** The fraction of recovered for various δ with η = 0.05, α = 0.3, α_0_ = 0.2.

Similarly, we also explore the impact of the rate of losing awareness δ on epidemic spreading. Figure 6 displays the evolution of ρ^*I*^(*t*) and ρ^*V*^(*t*) for four typical δ = 0.02, 0.2, 0.5, and 0.8, respectively. From Figure 6A, the fraction of Infected individuals raises when larger δ is applied. In addition, it can be found that the influence of increasing δ is similar to reducing η, α and α_0_. From Figure 6B, we can find that the increase of δ negatively affects the vaccinating process, i.e., the fraction of vaccinated individuals reduces with the raise of δ. The fraction of infected individuals presents different trends. This is because for lager δ, individuals would prefer not to take any measures to preserve themselves, and are more likely to be infection.

Furthermore, we analyze the impact of various δ on the fraction of individuals who aware/recovered by different ε in Figures 6C,D. It is obvious that the fraction of recovered individuals will enhance if greater δ is applied. This is because the increase of δ has a negative effect in the information diffusion process, decreasing aware-susceptible individuals, and incurs a higher infection probability and recovered individuals. Those phenomena once again illustrate the importance of taking effective preventative measures for the people who are aware of epidemic. Considering that the epidemic had been effectively controlled by Chinese government prompted strict measures (29–31), the spread of COVID-19 had been reduced to sporadic local outbreaks in China.

For example, in July (between the 27 June and 13 July, 2021), the individuals thought ‘not many people contract the disease, so the chances are low for me too’ they didn't think they need a vaccine in China. Thus, individuals lacked sufficient information, and lose his/her alertness, resulting in their reluctance to get vaccinated and subsequently the low vaccination rates.

**Figure 7:**
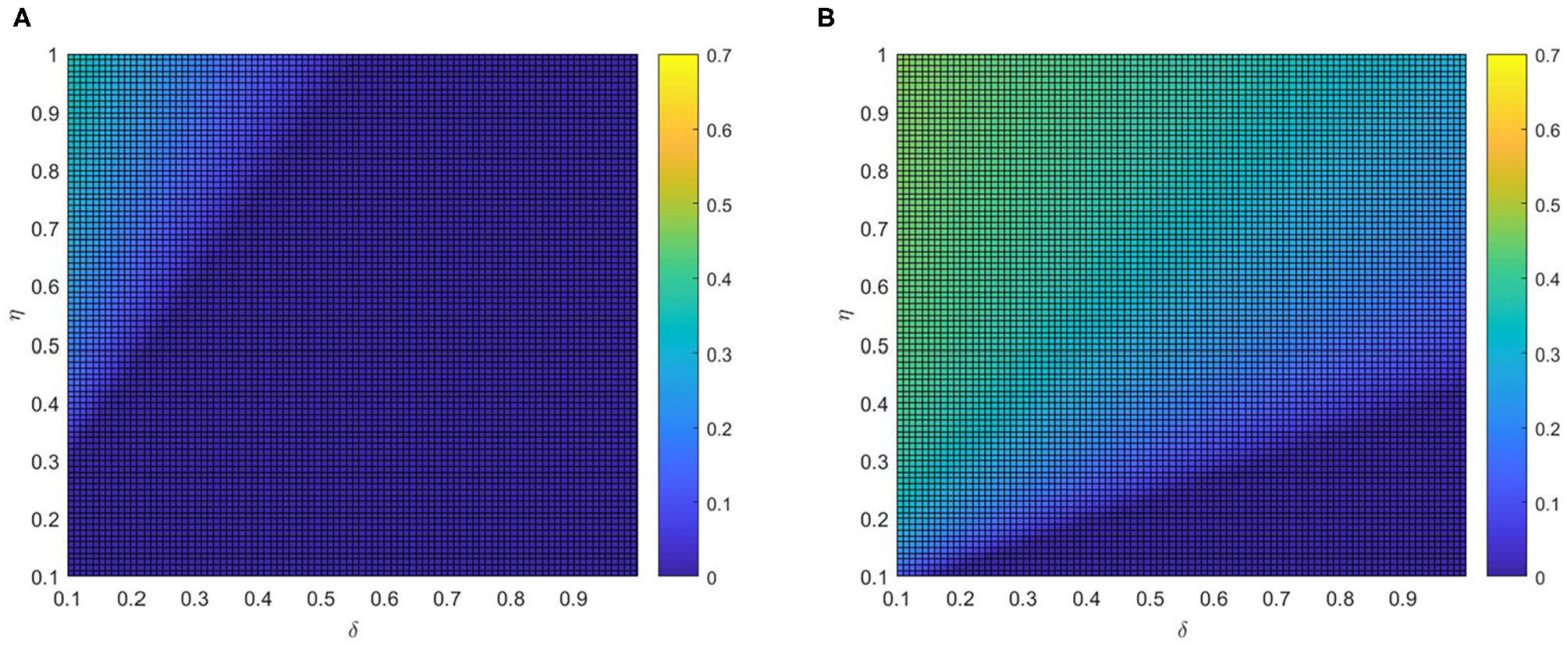
The fraction ρ^*V*^ of vaccinated individuals in the stationary state, where ε = 0.05, λ = 0.2 and ρ = 0.05. **(A)** α =0.2 and **(B)** α =0.8.

**Figure 8:**
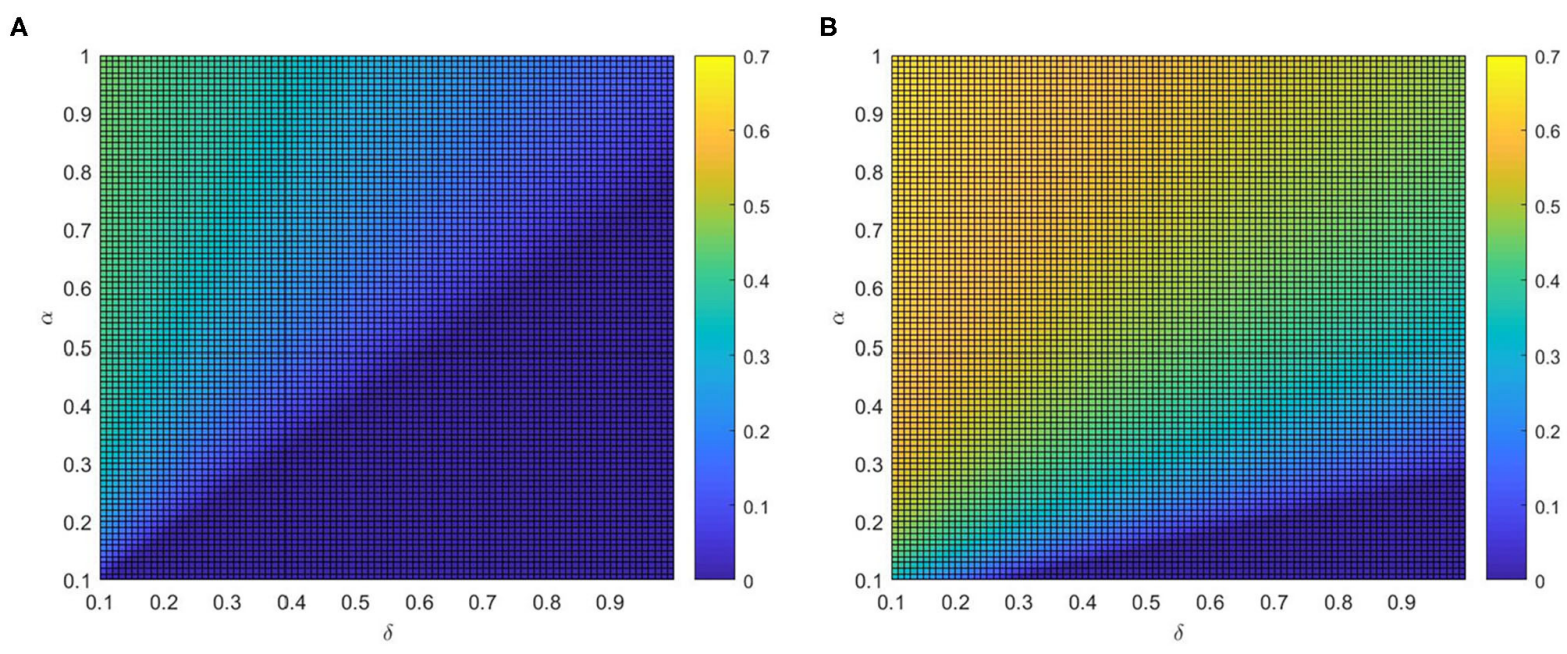
The fraction ρ^*V*^ of vaccinated individuals in the stationary state, where ε = 0.05, λ = 0.2 and ρ = 0.05. **(A)** η = 0.2 and **(B)** η = 0.8.

After that, the influence of various combinations of (η, δ) and (α, δ) on critical variate (i.e., fraction of vaccinated individuals) are farther studied. Comparing panel Figures 7A,B, when the local awareness ratio α is a higher one, the impact of the global awareness ratio η on fraction of vaccinated is greater for a fixed δ. In Figure 8, a similar trend is observed, however, the difference between Figures 7A,B shows more significant than that between Figures 8A,B, which implies that ρ^*V*^ is more remarkably influenced by the value of η than α. The main reason is that individuals are used to the gain and loss of awareness brought by neighborhood awareness and the impact is not as great as the occasional authoritative government information, especially, when the epidemic entered a controlled normalization process. Moreover, we also can find that the final vaccinated size, for the smaller awareness forgetting rate δ, is increasing faster due to the increase of η and α which is in Figures 7B, 8B. This phenomenon further demonstrate that global information is more effective than local information dissemination when the inertia of population behavior is very low (awareness decay).

For example, in mid-May (between 20 April and 15 May, 2021), even with COVID-19 largely contained, China continued to implement the harshest lockdown measures to combat the COVID-19 spreading when local outbreaks popped up. Such measures, along with a renewed fear of catching the virus, were strong incentives for getting vaccinated. At the same time, to expand vaccination coverage, health education and conversation from authoritative sources were influential ways to assuage public concernment about vaccine safety (32) (i.e., experts Zhang Wenhong and Zhong Nanshan also actively advocated the injection of COVID-19 vaccines). Thus, measures implemented, together with advocacy from experts, prompted the augment of the global information and encouraged aware people to get vaccinated, inducing the ascending rates of vaccination.

**Figure 9:**
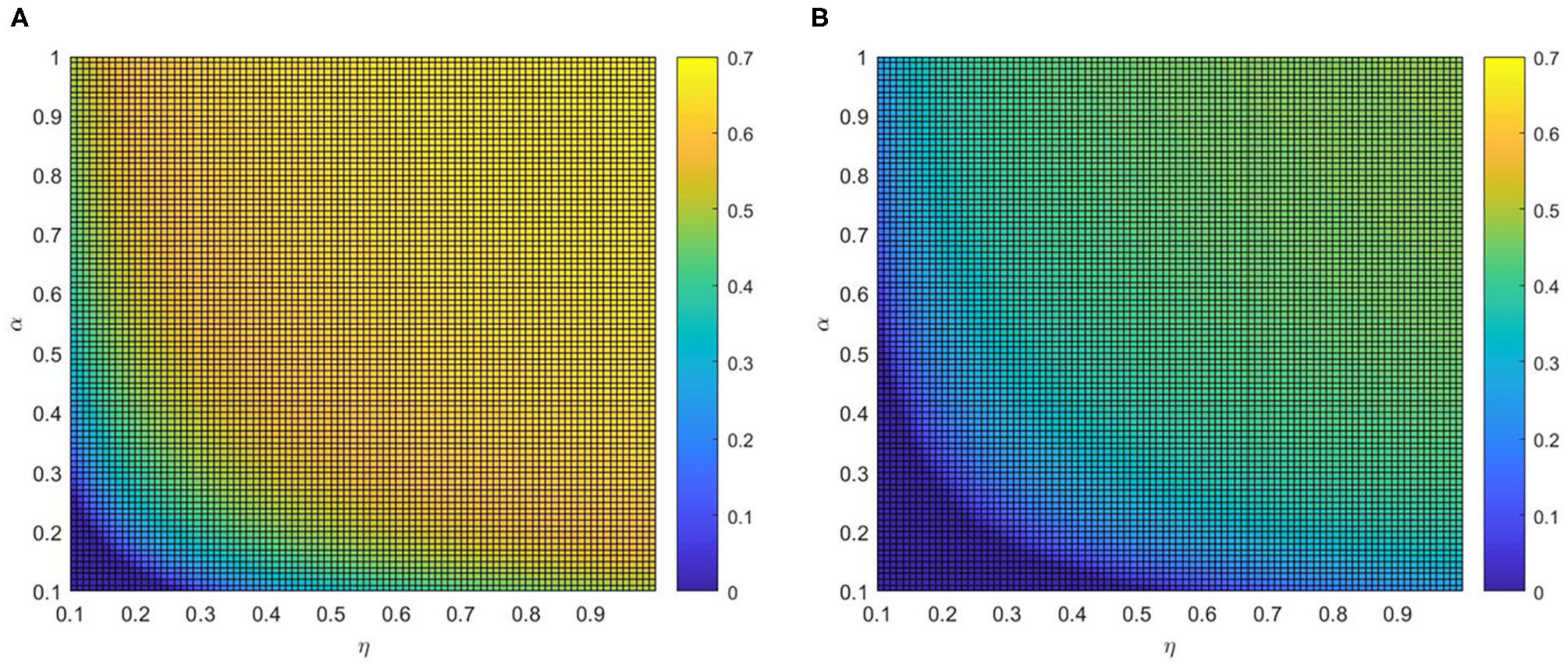
The fraction ρ^*V*^ of vaccinated individuals in the stationary state, where ε = 0.05, λ = 0.2 and ρ = 0.05. **(A)** δ = 0.2 and **(B)** δ = 0.8.

We investigate the effects of varying η and α on the vaccination coverage in Figure 9. From Figure 9A, it is clear that there exists an optimal area of ρ^*V*^(∞) in the parameter plane of η and α. The final size of vaccinated individuals ρ^*V*^(∞) will become small when η and α are out of the optimal region. Meanwhile, it is the normal case where ρ^*V*^(∞) increases with both η and α if both η and α are relatively large. And comparing Figure 9A with Figure 9B, awareness decay higher will decrease not only the fraction of vaccinated individuals but also the rate of growth. This phenomenon illustrates that information greatly affects disease prevention and reminds people to take protective measures against diseases, the higher global information η and local information α, the larger possibility for the individuals to take vaccination. Hence, mixing patterns in information can prominently influenced the fraction of vaccinated individuals. In real life just as the phenomenon, media activities promote discussion that can bring about behavioral altering. Infected individuals may learn from their experience and further convey this to their family and friends. This can be considered an important conclusion because it opens up the possibility to tune or optimize the response to limit the potential of epidemic spreading.

**Figure 10:**
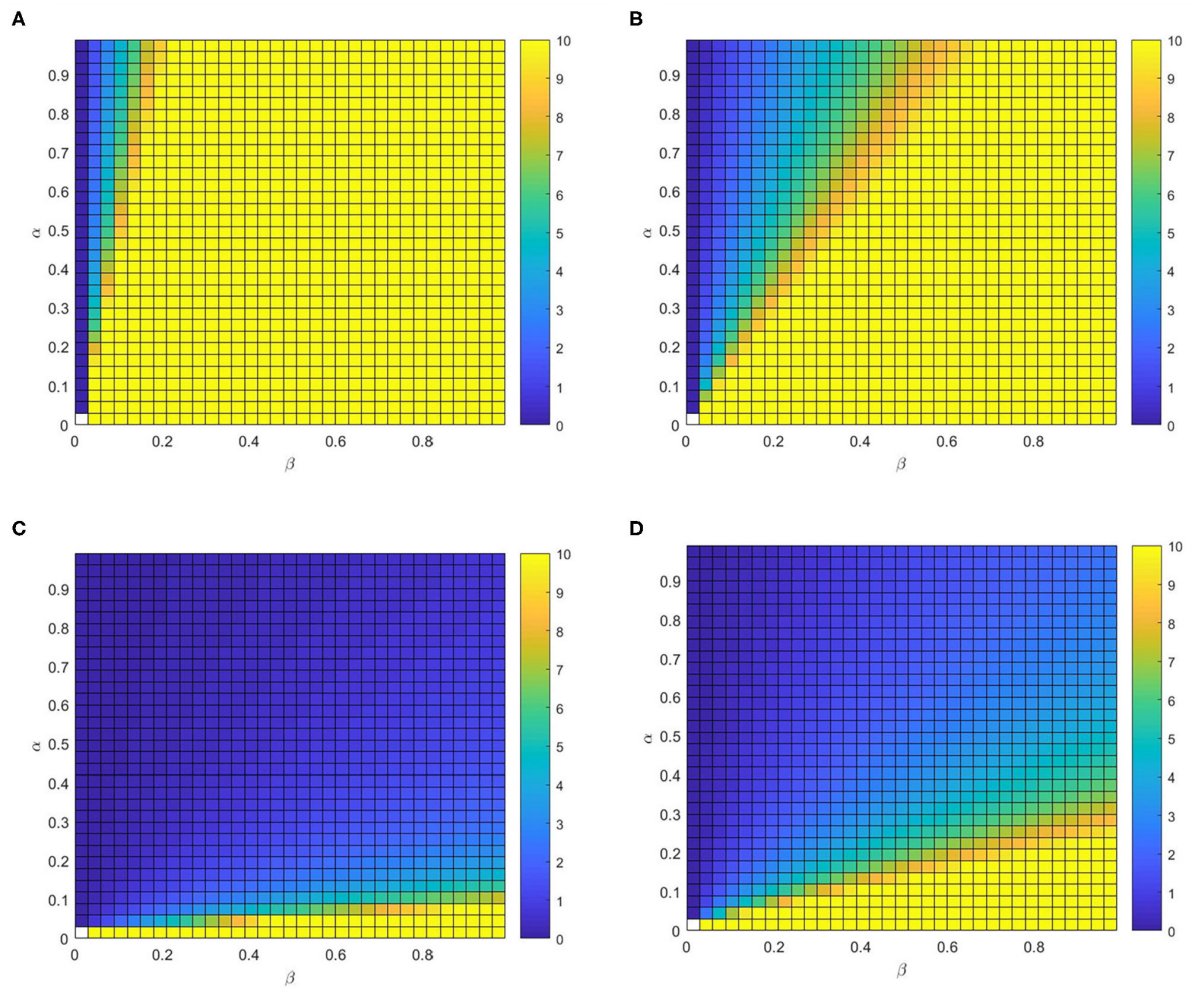
The control reproduction number *R*_*V*_. Full phase diagram α − β for the identical multiplex depicted before. **(A)** η = 0.02, δ = 0.8, **(B)** η = 0.02, δ = 0.2, **(C)** η = 0.4, δ = 0.2, and **(D)** η = 0.4, δ=0.8.

Finally, we further study the full phase diagram (α − β) to methodically explore the influence of η and δ on the *R*_*V*_ in Figure 10. Generally, we can find that *R*_*V*_ is not affected by α if β is less than the epidemic threshold, due to infectious disease will disappeared by itself. Once β is higher than the epidemic threshold, *R*_*V*_ reduces with α for different values of η or δ. More precisely, it can notice that *R*_*V*_ is not significantly affected by the varying of δ by contrast Figure 10A with Figure 10B (or contrast Figure 10C with Figure 10D). Similarly, it is obvious that *R*_*V*_ reduces with η, particularly for the large value of β by contrast the Figure 10A with Figure 10D (or contrast Figure 10B with Figure 10C).

Consequently, the best response to control the infectious disease spread is making *R*_0_ smaller by encouraging individuals to prevent themselves from infection which means increasing η and α. The spread of epidemic can be controlled if we have more susceptible individuals choosing to be aware.

## Conclusion

The research provided scientific evidence for the complicated interaction between awareness information and individuals vaccination behaviors in epidemic dynamics and control, highlighted the emphasis of authoritative and local information to promote behavioral changes and unrevealed awareness fading resulting in low vaccination rate. The study could be effectively implemented even with the multitude of sequential waves observed in the case of COVID-19. As of revised paper submission (23 December 2021), a medium-scale outbreak caused by the Delta variant in provinces Zhejiang and Shaanxi prompted authorities to institute heightened restrictions in multiple cities. It also promoted individuals to take a COVID-19 vaccine booster shot immediately to combat the virus variant. Because individuals were more likely to gain the awareness of vaccination once they received strong stimulations of updated local information about the reported number of infections, resulting in the spurt of vaccination coverage growth. Shortly before the confirmed local outbreak, demand for COVID-19 vaccine had slowed in months, presenting a worrying trend that could delay achievement of herd immunity. Since China continued to implement the harshest lockdown measures, most individuals took it for granted that the chance of infection was low and they didn't need vaccinations to protect themselves. It reflected that individuals lacked sufficient local epidemic information and lost his/her alertness, subsequently resulted in the decrease of vaccination rates. The study suggests that the government need to provide the sustained health education and communication to alleviate awareness decay, and prompt individuals to adopt spontaneous behavioral responses in order to protect themselves to be awareness individuals again.

## Data Availability Statement

The original contributions presented in the study are included in the article/supplementary material, further inquiries can be directed to the corresponding author.

## Author Contributions

CZ proposed framework and implemented the simulation experiments. FZ and YL contributed to model building, data analysis, and writing the manuscript. All authors contributed to the article and approved the submitted version.

## Funding

This work was supported by National Social Science Foundation of China (Grant No. 21BGL298).

## Conflict of Interest

The authors declare that the research was conducted in the absence of any commercial or financial relationships that could be construed as a potential conflict of interest.

## Publisher's Note

All claims expressed in this article are solely those of the authors and do not necessarily represent those of their affiliated organizations, or those of the publisher, the editors and the reviewers. Any product that may be evaluated in this article, or claim that may be made by its manufacturer, is not guaranteed or endorsed by the publisher.
